# Radiosensitization by Gold Nanoparticles: Impact of the Size, Dose Rate, and Photon Energy

**DOI:** 10.3390/nano10050952

**Published:** 2020-05-17

**Authors:** Kirill V. Morozov, Maria A. Kolyvanova, Maria E. Kartseva, Elena M. Shishmakova, Olga V. Dement’eva, Alexandra K. Isagulieva, Magomet H. Salpagarov, Alexandr V. Belousov, Victor M. Rudoy, Alexander A. Shtil, Alexander S. Samoylov, Vladimir N. Morozov

**Affiliations:** 1 State Research Center-Burnasyan Federal Medical Biophysical Center of Federal Medical Biological Agency, 123098 Moscow, Russia; morozov.kv15@physics.msu.ru (K.V.M.); kolyvanova@physics.msu.ru (M.A.K.); kia2303@yandex.ru (A.K.I.); magometonco@mail.ru (M.H.S.); belousovav@physics.msu.ru (A.V.B.); shtilaa@yahoo.com (A.A.S.); asamoilov@fmbcfmba.ru (A.S.S.); 2 Department of Physics, Lomonosov Moscow State University, 119234 Moscow, Russia; 3 Emanuel Institute of Biochemical Physics, Russian Academy of Sciences, 119334 Moscow, Russia; 4 Frumkin Institute of Physical Chemistry and Electrochemistry, Russian Academy of Sciences, 119071 Moscow, Russia; maryakar@mail.ru (M.E.K.); alena_shishmakova@mail.ru (E.M.S.); redmoun@mail.ru (O.V.D.); dema_ol@mail.ru (V.M.R.); 5 Gause Institute of New Antibiotics, 119021 Moscow, Russia; 6 Blokhin National Medical Research Center of Oncology, 115478 Moscow, Russia

**Keywords:** radiosensitizers, gold nanoparticles, X-ray, dose rate, plasmid DNA, radiotherapy

## Abstract

Gold nanoparticles (GNPs) emerged as promising antitumor radiosensitizers. However, the complex dependence of GNPs radiosensitization on the irradiation conditions remains unclear. In the present study, we investigated the impacts of the dose rate and photon energy on damage of the pBR322 plasmid DNA exposed to X-rays in the presence of 12 nm, 15 nm, 21 nm, and 26 nm GNPs. The greatest radiosensitization was observed for 26 nm GNPs. The sensitizer enhancement ratio (SER) 2.74 ± 0.61 was observed at 200 kVp with 2.4 mg/mL GNPs. Reduction of X-ray tube voltage to 150 and 100 kVp led to a smaller effect. We demonstrate for the first time that the change of the dose rate differentially influences on radiosensitization by GNPs of various sizes. For 12 nm, an increase in the dose rate from 0.2 to 2.1 Gy/min led to a ~1.13-fold increase in radiosensitization. No differences in the effect of 15 nm GNPs was found within the 0.85–2.1 Gy/min range. For 21 nm and 26 nm GNPs, an enhanced radiosensitization was observed along with the decreased dose rate from 2.1 to 0.2 Gy/min. Thus, GNPs are an effective tool for increasing the efficacy of orthovoltage X-ray exposure. However, careful selection of irradiation conditions is a key prerequisite for optimal radiosensitization efficacy.

## 1. Introduction

The ability of certain chemical compounds to enhance the biological effect of ionizing radiation had long been known [1]. However, the search for new radio modifiers for radiation therapy (RT) remains important [2,3,4]. Nanoparticles (NPs) of metals with the high atomic number (*Z*) are a new promising class of antitumor radiosensitizers both for classic photon and particle RT [5,6,7,8]. Gold nanoparticles (GNPs) are particularly attractive since they increase the efficacy of ionizing radiation in various biological systems including molecular solutions [9,10], cell-based models [11,12], and in vivo [13,14].

The shape and the size of GNPs as well as their surface properties may vary widely [15,16,17]. This diversity allows for design of GNPs potentially applicable in RT of various tumors. On the other hand, since the cornerstone of RT is the accuracy of dose delivery and treatment predictability, it is exceptionally important to identify the most efficient regimens of irradiation in the presence of GNPs. However, nano-radiosensitizers are rather delicate systems. Even minor changes in their characteristics may affect their interaction with cells [18,19] as well as the physicochemical aspects of radiosensitization [20,21,22,23,24,25,26] ultimately leading to significant differences in the magnitude of the effect of NPs [27,28,29,30].

In addition to the rational design of GNPs, a key factor is the selection of optimal irradiation conditions that provide the required efficacy of radiosensitization. Different photon sources (X-ray therapy machines, electron accelerators, and brachytherapy sources) with the energy range of 30 keV–25 MeV are widely used in the clinic. Although GNPs exhibit radiosensitization in combination with many photon sources [31], the efficacy significantly depends on the radiation energy and its spectral composition [9,32,33,34]. Even in the case of synchrotron exposure, the dependence of GNPs’ radiosensitization effect on radiation energy is not completely clear [35,36].

These considerations justified the search for optimal irradiation parameters that would provide the maximum radiosensitization effect of spherical GNPs with a given size and surface chemistry. We exposed the pBR322 plasmid to X-ray photons as a model of radiation-induced DNA damage. The dependence GNPs’ radiosensitization efficacy on the conditions of irradiation was studied by varying the dose rate and voltage on the X-ray tube. To evaluate the radio-sensitizing efficacy, the values of the sensitizer enhancement ratio (SER), mono-dose enhancement factor (EF), and physical dose enhancement factor (DEF) were calculated.

## 2. Materials and Methods

To synthesize GNPs, we used chloroauric acid trihydrate (≥99%, Acros Organics, Geel, Belgium) and sodium citrate (99%, Sigma-Aldrich, St. Louis, MO, USA). Distilled water additionally deionized with an Arium 611 purification system (Sartorius, Göttingen, Germany) was used as a solvent. Spherical GNPs were synthesized according to a multi-step protocol [37]. First, the seed NPs (Sample 1) were obtained by injecting a solution of HAuCl_4_ (1 mL, 25 mM) into the boiling solution of sodium citrate (150 mL, 2.2 mM) under vigorous stirring. After boiling for 10 min, the reaction system was cooled down to 90 °C, and the stepwise growth of Au seeds was carried out. In so doing, two portions of HAuCl_4_ solution (1 mL, 25 mM) were injected into the system 30 min apart. The obtained dispersion (Sample 2) was diluted by replacing 55 mL of its volume by the same volume of 2.2 mM sodium citrate. This two-step procedure of seed growth was repeated twice and yielded sample 3 and sample 4, respectively.

The size of GNPs was determined using a Technai G2 F-20 S-Twin TMP (FEI, Eindhoven, Netherlands) high-resolution transmission electron microscope (HRTEM) operating at an accelerating voltage of 200 kV. A droplet of aqueous GNP dispersion was placed onto a formvar-coated copper grid, exposed on air for 1 min, and then removed with the filter paper. HRTEM images were processed using ImageJ software (National Institutes of Health, Bethesda, MD, USA). In some cases, a Zetasizer Nano ZS dynamic light scattering (DLS) spectrometer (Malvern Panalytical, Malvern, United Kingdom) was used to determine the size of GNPs. The results obtained by HRTEM and DLS techniques were in good agreement.

Extinction spectra of Samples 1–4 were recorded on an Evolution 300 double-beam spectrophotometer (Thermo Electron Corp., Waltham, MA, USA) at the wavelength range of 250–800 nm.

The concentration of metal Au, *C*_Au0_, in the dispersion was estimated by the formula below [38].

*C*_Au0_ = 0.355*D*_390_ (1)

where *D*_390_ is the optical density of dispersion at 390 nm, corresponding to the region of Au inter-band transitions. Its value is determined only by the concentration of metal (*C*_Au0_) and does not depend on the particle size and shape.

**Figure 1:**
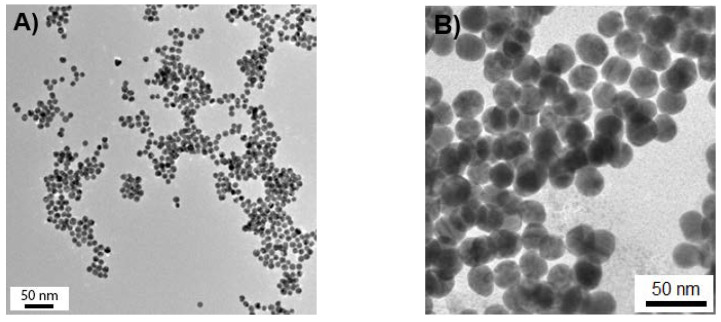
High-resolution transmission electron microscope (HRTEM) images of (**A**) sample 1 and (**B**) sample 4 of the prepared gold nanoparticles (GNPs).

Figure 1 shows typical HRTEM images of GNPs. The prepared particles show narrow size distributions. Their main characteristics are given in Table 1.

The number concentration of particles (i.e., [GNP]) was calculated by dividing *C*_Au0_ by the mass of one GNP that was estimated from the average diameter of GNPs and the density of bulk Au. For the convenience of comparing our data with the other literature date, the [GNP] values are given in nM and mL^−1^.

For experiments with the plasmid DNA, the dispersions of GNPs were concentrated by the following procedure. GNPs were precipitated by centrifugation (Hettich universal 320R centrifuge, Tuttlingen, Germany), and the supernatant was partly decanted to reach gold content 3 mg/mL (see Table 1). The precipitate was re-dispersed in the remaining volume of the supernatant using an ultrasonic bath. Aqueous solutions (25 µL, pH = 6.9–7.1) of pBR322 plasmid (4361 bp; Invitrogen, Waltham, MA, USA) were prepared in 0.5 mL polypropylene tubes (Eppendorf, Hamburg, Germany). DNA concentration was 0.04 mg/mL. The concentrations of GNPs ranged from 0.6 mg/mL to 2.4 mg/mL (three samples were used in each experiment). The binding of GNPs to DNA is a rather fast process [39]. Our preliminary experiments showed that the magnitude of radiosensitization did not depend on the incubation time within 5–120 min range (data not shown). Therefore, GNPs and DNA were co-incubated 30 min at room temperature prior to irradiation.

**Figure 2:**
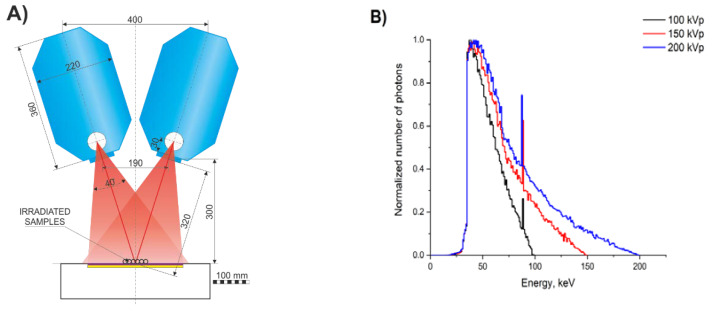
(**A**) Scheme of irradiation. (**B**) Spectra of RUST-M1 at various X-ray tubes voltages calculated using Monte-Carlo simulation: 100 kVp (black), 150 kVp (red), and 200 kVp (blue).

Irradiation was carried out on a RUST-M1 X-ray machine (Diagnostika-M, Moscow, Russia). The scheme of exposure is shown in Figure 2A. Radiation was generated by two simultaneously operating X-ray tubes RAP 220-5. The voltage on the X-ray tubes was 100, 150, or 200 kVp. In addition, 1.5 mm Al filter was used. The dose rate was 0.2–2.1 Gy/min. The exposed doses were 0–40 Gy with a 5 Gy increment. X-ray spectra calculated by the Monte-Carlo method using a Geant4 toolkit [40] are shown in Figure 2B. A detailed description of the methodology for calculating X-ray spectra has been presented in Reference [41].

DNA samples were centrifuged (11,000 rpm, 15 min) immediately after irradiation to remove GNPs. The supernatant was used for electrophoresis in 1% agarose gel (Tris base-sodium acetate-ethylenediaminetetraacetic acid sodium salt buffer pH 8.3, 90 min at 85 kV). Gels were stained with ethidium bromide (EtBr) and visualized by UV excitation (λ = 365 nm). The supercoiled (SC), circular (C), and linear (L) forms of plasmid DNA were detected. Quantitative analysis of electrophoregrams was performed using ImageJ software. Since the fluorescence intensity of EtBr bound to SC DNA is significantly lower than the intensities of EtBr complexes with C and L DNA forms, a correction factor of 1.7 for SC intensity was used [42,43].

SER values were calculated from fitting of the experimental dose-damage curves for the fixed DNA damage effect (i.e., % SC) by the formula below.

(2) $$\mathrm{SER}=\frac{D_{\mathrm{count}}}{D_{\mathrm{GNPs}}}$$

where *D*_cont_ is the dose for the observed effect in the control sample without GNPs and *D*_GNPs_ is the dose evoking the same effect in the presence of GNPs. The EF values [44] for a given radiation dose were calculated using the formula.

(3) $$\mathrm{EF}=\frac{\%\;\mathrm{SC}\;w/o\;\mathrm{GNPs}}{\%\;\mathrm{SC}\;\mathrm{with}\;\mathrm{GNPs}}$$

Physical DEF is defined as the ratio of the dose absorbed by the volume of interest in the presence of GNPs (*D*_2_) to the dose absorbed by the same volume in the absence of GNPs (*D*_1_).

(4) $$\mathrm{DEF}=\frac{D_{2}}{D_{1}}$$

If the absorbed dose *D*_1_ in substance 1 is known, then, under conditions of electronic equilibrium, the absorbed dose *D*_2_ in substance 2 at the same point of the irradiation field can be calculated by the equation below.

(5) $$D_{2}=\frac{{\left(µ/\rho \right)}_{2}}{{\left(µ/\rho \right)}_{1}}D_{1}$$

where (µ/*ρ*)_i_ is the photon mass absorption coefficient of the *i*-th substance. In the case of monochromatic radiation and a small volume of interest, the conditions of electronic equilibrium are satisfactory to determine the absorbed dose with a ≤10% error. In the case of a polychromatic source, Formula (5) takes the following form.

(6) $$D_{2}=\frac{\sum_{i}E_{i}n_{i}{\left(\frac{µ\left(E_{i}\right)}{\rho }\right)}_{2}}{\sum_{i}E_{i}n_{i}{\left(\frac{µ\left(E_{i}\right)}{\rho }\right)}_{1}}D_{1}=\frac{\bar{{\left(\frac{µ}{\rho }\right)}_{2}}}{\bar{{\left(\frac{µ}{\rho }\right)}_{1}}}D_{1}=\bar{{\left(\frac{µ}{\rho }\right)}_{1}^{2}}D_{1}$$

In Formula (6), *n*_i_ stands for the number of photons with energy *E*_i_ and summation is performed over the energies of all photons emitted in the decay (including X-ray radiation due to the electron shell transitions). Thus, we obtain the following expression for DEF.

(7) $$\mathrm{DEF}=\bar{{\left(\frac{µ}{\rho }\right)}_{1}^{2}}$$

The values of the photon mass absorption coefficients were taken from Reference [45]. For a substance mixed of various chemical elements, the mass absorption coefficient can be calculated by the equation below.

(8) $$\frac{{µ}_{\mathrm{en}}}{\rho }=\underset{i}{\sum }w_{i}{\left(\frac{{µ}_{\mathrm{en}}}{\rho }\right)}_{i}$$

where ${\left(\frac{{µ}_{\mathrm{en}}}{\rho }\right)}_{i}$ is the energy absorption coefficient of the *i*-th element in the mixture and *w*_i_ is the weight fraction of this element in the mixture.

## 3. Results and Discussion

In the present paper, we investigated GNPs radiosensitization in combination with kilovoltage X-ray radiation. Although X-ray RT machines are significantly less frequent than linear accelerators (e.g., according to the International Atomic Energy Agency (IAEA) for 2020: 31 vs. 67 in Sweden, 83 vs. 447 in Russia, 54 vs. 348 in United Kingdom, 7 vs. 3827 in USA [46]), they are in use for the treatment of superficial neoplasms like skin cancer as well as various non-tumor diseases [47]. X-ray radiation sources are also used in brachytherapy and intraoperative RT [48].

Optimization of the use of nano-radiosensitizers for given clinical conditions is critical for therapeutic efficacy of RT [49]. We investigated optimal exposure conditions for given GNPs. One parameter was used as a variable at each step, that is, either GNP concentration or the dose rate, or X-ray tube voltage. When the highest radiosensitization was achieved, the value of the respective parameter was fixed, and the experiment proceeded to the next step.

**Figure 3:**
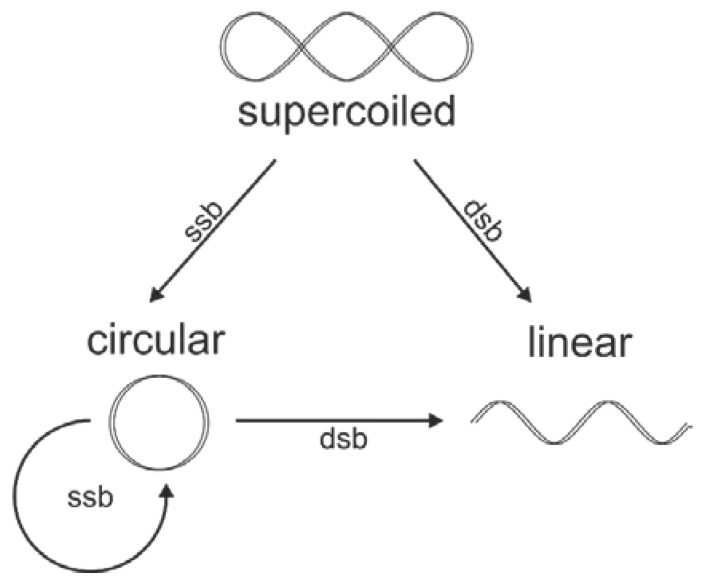
Schematic representation of the radiation induced topological changes in plasmid DNA [42]. The ssb and dsb correspond to single-strand and double-strand breaks, respectively.

The plasmid DNA as a target of ionizing radiation is widely used in studies of radio-modifiers including high-*Z* NPs [9,35,50,51,52]. This model is convenient for unraveling the physical and physicochemical mechanisms of GNPs radiosensitization and for quantifying the radiation induced damage of bio-macromolecules. As shown in Figure 3, the radiation induced single-stranded (ssb) and double-stranded (dsb) breaks of the initially SC DNA, which led to the formation of C or L forms of DNA, respectively. These topological isomers of DNA can be identified by different electrophoretic mobility in a gel.

**Figure 4:**
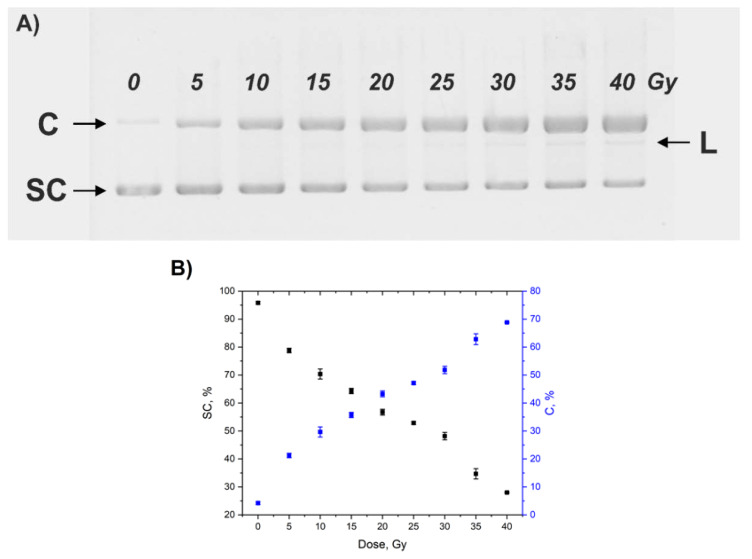
(**A**) Electrophoregram of pBR322 DNA irradiated with 0–40 Gy. SC, supercoiled. C, circular. L, linear forms. (**B**) Dependences of the amounts of SC and C forms of pBR322 DNA on the dose of X-ray irradiation. Error bars are mean confidence intervals calculated by three parallel independent measurements for *α* = 0.05.

The typical electrophoregram of pBR322 DNA exposed to X-ray (0–40 Gy) is shown in Figure 4A. In the non-irradiated sample (lane 0) >95% of the plasmid DNA is in a SC form. X-ray irradiation led to a dose-dependent decrease in the amount of the SC form and a concomitant increase in the C form. The L form is detectable only at doses over ~25 Gy but its amount did not exceed 5%. Plots of dose dependences of the content of SC and C DNA forms are shown in Figure 4B.

**Figure 5:**
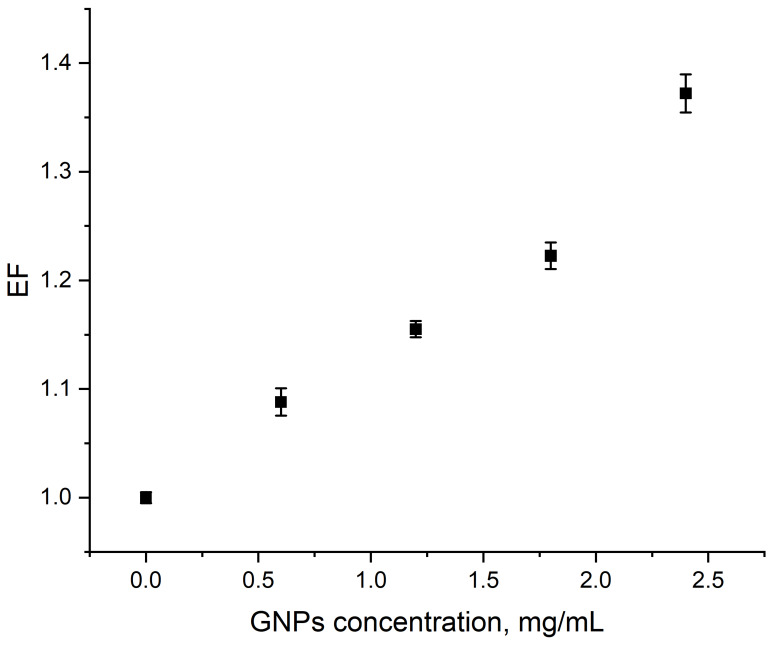
Dependence of enhancement factor (EF) on the concentration of 26 nm GNPs (5 Gy, 200 kVp, 0.2 Gy/min). The GNP:DNA ratio in the samples ranged from 0.075 to 1.5 particles per one DNA molecule. Error bars are mean confidence intervals calculated by three parallel independent measurements for *α* = 0.05.

Irradiation of the plasmid in the presence of GNPs led to an increase in radiation-induced damage. The dependence of EF on GNP concentration (*d* = 26 nm) in the range of 0.6–2.4 mg/mL is shown in Figure 5. Total dose is 5 Gy, the tube voltage is 200 kVp, and the 0.2 Gy/min dose rate is fixed. The mass ratio of DNA and GNPs ranged from 1:3 to 1:60, i.e., from 0.075 to 1.5 particles per one DNA molecule. At these concentrations, GNPs alone did not alter plasmid DNA conformation without radiation (data not shown). EF values increased from 1.09 ± 0.01 to 1.37 ± 0.02 along with elevated GNP concentration. The shape of the concentration dependence is similar to the one reported earlier [9].

The impact of the radiation dose rate is an underestimated issue in high-Z NPs radiosensitization. Changes in the dose rate noticeably affected the magnitude of radiosensitization by metal and metal oxide NPs [53,54,55]. The yield of the model reaction of coumarin carboxylic acid hydroxylation under X-ray irradiation was dramatically enhanced in the presence of gold and platinum NPs [53]. In this case, for 7 nm GNPs, an increase in the dose rate from 3.3 Gy/min to 20 Gy/min led to a several-fold enhancement. At the same time, an increased production of highly fluorescent 7-hydroxycoumarin in the presence of 32.5 nm GNPs, showing an increase in hydroxyl radicals’ production, was observed along with a decrease in the dose rate from 936 Gy/min to 36 Gy/min [54]. However, the ranges of dose rate values (>6–10 Gy/min) investigated in References [53,54] are not widely used in the clinical practice. The effect of the dose rate was even more complex for cell models [55]. The magnitude of radiosensitization by the dextran-coated iron oxide NPs in HeLa and MCF-7 cells rose with the increase in the dose rate from 40 cGy/min to 370 cGy/min. The maximum effect for HeLa cells was achieved at 370 cGy/min and 80 µg/mL GNPs whereas irradiation of MCF-7 breast adenocarcinoma cells without NPs gave the same effect at 40 cGy/min. At the same time, the absence of the effect of the dose rate on the yield of radicals in aqueous methanol solutions containing 84 nm HfO_2_ NPs has been shown [56].

**Figure 6:**
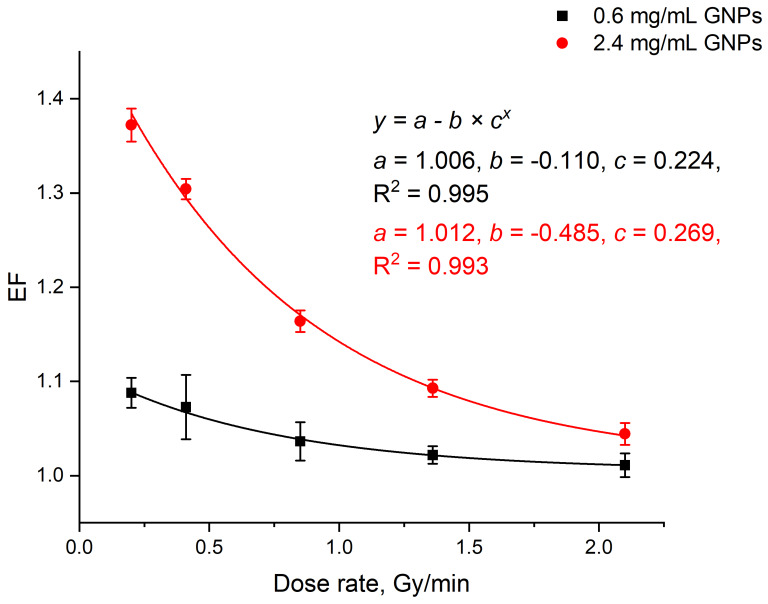
Dependence of EF on the dose rate at 0.6 mg/mL (black) and 2.4 mg/mL (red) GNPs (d = 26 nm, 5 Gy, and 200 kVp). Error bars are mean confidence intervals calculated by three parallel independent measurements for *α* = 0.05.

In our experiments, at a fixed dose of 5 Gy and a voltage of 200 kVp, the following dose rates were tested: 0.2 Gy/min, 0.41 Gy/min, 0.85 Gy/min, 1.36 Gy/min, and 2.1 Gy/min. This range of the dose rates matches the clinical conditions of external kilovoltage X-ray RT and electronic brachytherapy. The change in the dose rate did not affect DNA damage in the absence of GNPs. In contrast, in the presence of GNPs, a noticeable change in the magnitude of this effect was observed. Figure 6 shows the dependence of EF on the irradiation dose rate for GNP concentrations of 0.6 mg/mL and 2.4 mg/mL. At 2.1 Gy/min, which is the highest tested dose rate, 26 nm GNPs showed the weakest effect: EF = 1.01 ± 0.01 and 1.04 ± 0.01 at 0.6 mg/mL and 2.4 mg/mL, respectively. A gradual decrease in the dose rate to 0.2 Gy/min, similarly to previous results [54], led to an increased DNA damage of EF = 1.09 ± 0.02 and 1.37 ± 0.02 at the same GNP concentrations. Thus, in this model, the highest radiosensitization by 26 nm GNPs was achieved at the lowest dose rate.

**Figure 7:**
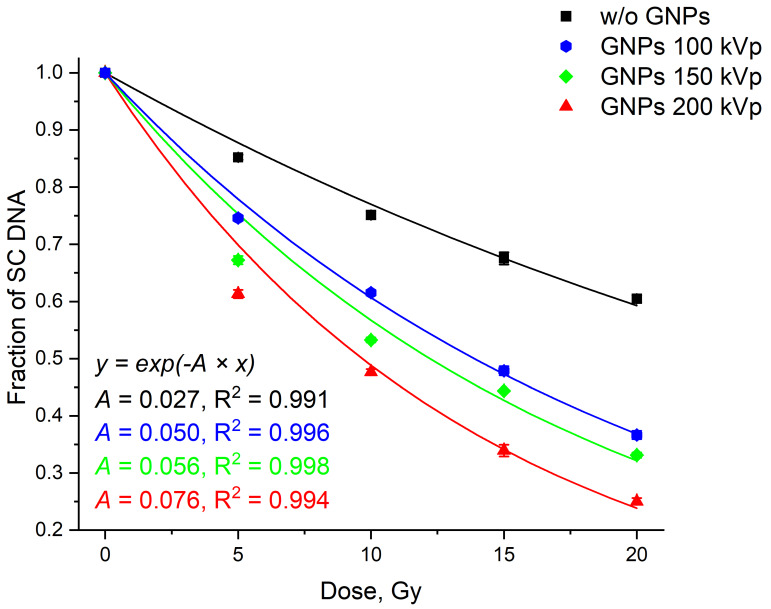
Dependencies of DNA damage on the irradiation dose at a different voltage on the X-ray tube: without GNPs (200 kVp, black) and in the presence of 2.4 mg/mL GNPs (d = 26 nm) at 100 kVp (blue), 150 kVp (green), and 200 kVp (red). The GNP:DNA ratio in the samples was 1.5 particles per one DNA molecule. Error bars are mean confidence intervals calculated by three parallel independent measurements for *α* = 0.05.

The voltage on the X-ray tube may also influence the efficacy of GNPs radiosensitization. The amounts of the SC DNA as a function of the dose in the range of 0 to 20 Gy for 100, 150, and 200 kVp voltage values at a fixed dose rate of 0.2 Gy/min (26 nm GNP, 2.4 mg/mL, i.e., 1.5 GNPs per one DNA molecule) are shown in Figure 7. The control experiments without GNPs shown no statistically significant differences in the damage of plasmid DNA caused by the changes in voltage on the X-ray tube. At the same time, the increase in the voltage on the X-ray tube significantly enhances the GNPs radio-sensitizing effect. Experimental data fitting with the exponential model was carried out according to Reference [57]. Within this model, SER did not depend on the effect magnitude (i.e., fraction of SC form).

Values of SER and EF (Table 2 and Table 3) were also calculated by fitting the results using Formulas (2) and (3). The most pronounced radio-sensitization as determined by SER 2.74 ± 0.61 was achievable with 2.4 mg/mL GNPs, 200 kVp, and 0.2 Gy/min (red curve in Figure 7).

Next, we studied whether these irradiation conditions are optimal for other sizes of GNPs. The impact of the particle size on the magnitude of radiosensitization by GNPs is known even though the experimental data differ qualitatively. In Reference [9], the larger GNPs gave a greater radiosensitization. However, an increase in the particle size led to an increase in the internal absorption of secondary radiation [20]. Furthermore, 5-nm GNPs gave greater dose enhancement factors of double-strand breaks of DNA than 20-nm particles [50]. Misawa et al. [21] showed that the magnitude of the superoxide radical yield decreased with the increased size of GNPs.

**Figure 8:**
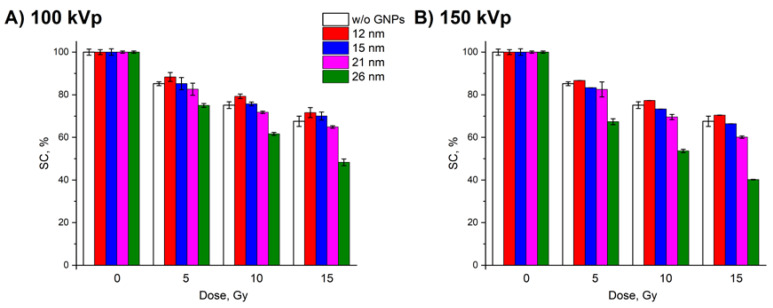

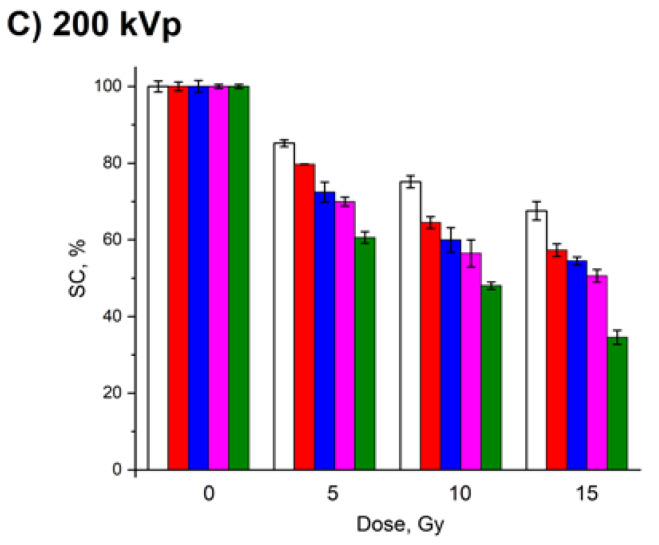
Effect of the X-ray tube voltage on the amount of SC DNA (relative to control samples): 100 kVp (**A**), 150 kVp (**B**), and 200 kVp (**C**). No GNPs (white). 2.4 mg/mL GNPs with a size of 12 nm (red), 15 nm (blue), 21 nm (magenta), and 26 nm (green). The GNP:DNA ratios in the samples were 15.3 (12 nm), 7.8 (15 nm), 2.8 (21 nm), and 1.5 (26 nm) particles per one DNA molecule. Error bars are mean confidence intervals calculated by three parallel independent measurements for *α* = 0.05.

Figure 8 shows the dose dependencies of the SC fraction after DNA irradiation in the presence of GNPs of various diameters for different X-ray tube voltages and a dose rate of 0.2 Gy/min. The trend observed for 26-nm GNPs was similar for other particle sizes. The increased voltage led to an increased radiosensitization. The maximal effect was observed at 200 kVp. The radiosensitization efficacy also increased with GNP size. The weakest radiosensitization was demonstrated by 12-nm GNPs. The greatest efficiency corresponded to 26-nm GNPs. At 200 kVp voltage, statistically significant differences for 15-nm and 21-nm GNPs were observed only at 15 Gy where 21-nm GNPs showed a slightly higher effect.

**Figure 9:**
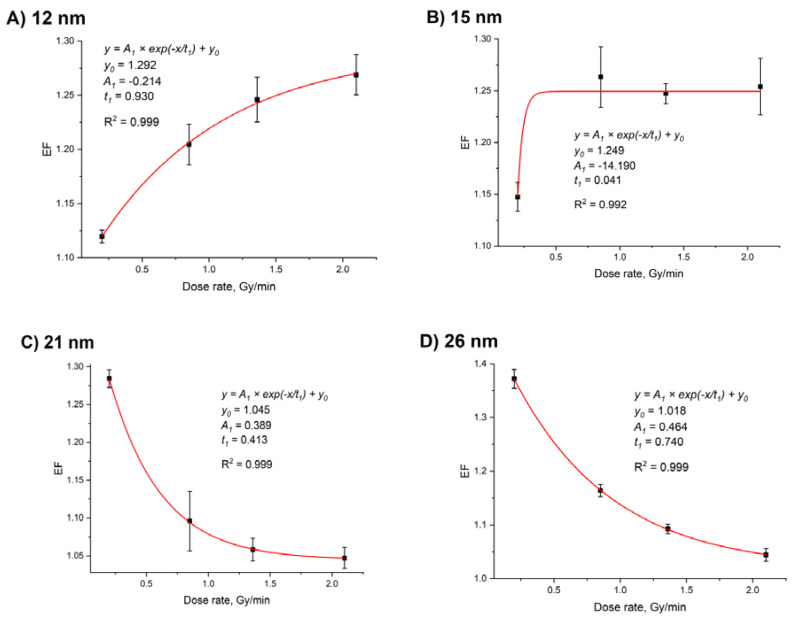
EF as a function of the dose rate for GNPs with a size of 12 nm (**A**), 15 nm (**B**), 21 nm (**C**), 26 nm (**D**) at a fixed dose (5 Gy) and tube voltage (200 kVp). Fitting was performed according to the exponential model. The GNP:DNA ratios in the samples were 15.3 (12 nm), 7.8 (15 nm), 2.8 (21 nm), and 1.5 (26 nm) particles per one DNA molecule. Error bars are mean confidence intervals calculated by three parallel independent measurements for *α* = 0.05.

In the present study, the greatest radiosensitization corresponded to the largest GNP size at a fixed dose rate of 0.2 Gy/min. However, variation of the dose rate may change this trend. To verify this assumption, we performed, for the first time, systematic experiments on the impact of the dose rate on radiosensitization by GNPs of various sizes (Figure 9). For 12-nm GNPs, an increase in the dose rate from 0.2 Gy/min to 2.1 Gy/min led to a ~1.13-fold increased radiosensitization. There were no statistically significant differences in radiosensitization efficacy of 15-nm GNPs along with the dose rate variations from 0.85 Gy/min to 2.1 Gy/min. For 21-nm and 26-nm GNPs, an increased radiosensitization was observed with the decrease in the dose rate from 2.1 Gy/min to 0.2 Gy/min. Thus, when the GNP size increased, the slope of the curve reversed from positive for 12 nm and 15 nm GNPs to negative for 21 nm and 26 nm GNPs. Thus, at 0.2 Gy/min dose rate, the largest (26 nm) GNPs showed the best radiosensitization while 12 nm and 15 nm GNPs were the most efficient at 2.0 Gy/min. Changes in the dose, voltage, and GNPs concentration influenced EF values only. The slope of the curves remained the same.

The influence of dose rate on the magnitude of radiosensitization cannot be explained by physical processes alone, but rather has a physicochemical/chemical nature. Clarification of the reasons for this effect is one of our next goals. Nevertheless, it can be assumed that such variability indicates the presence of several mechanisms of the GNPs’ radiosensitization depending on the specific conditions of the experiment. In this regard, we note that the multidirectional trends of the radiosensitization effect dependence on dose rate were also observed in References [53,54] (see above). Their authors discussed the different mechanisms of the radio-enhancement effect. According to Reference [50], reactions involving free radicals occur directly on the NP surface and the yield of products strongly depends on the nature of the NP material. At the same time, according to Reference [54], formation of hydroxyl radicals under the radiation exposure occurs in a structured water layer near the GNP surface in which intermolecular hydrogen bonds are lengthened and weakened.

A significant part of experimental studies on high-*Z* NPs radiosensitization has been performed with X-ray irradiation. Commonly, only the X-ray tube voltage is given whereas data on the anode material, filter characteristics, and the tube spectrum are less available. Nevertheless, the spectral composition of radiation significantly affects radiosensitization by NPs [34,58]. The highest radiosensitization enhancement factor was achieved with a lower X-ray tube voltage: 1.66 (105 kVp) vs. 1.43 (220 kVp) [33]. A similar trend was reported in Reference [32]: 24.6 (80 kVp) vs. 2.2 (150 kVp). Theoretical calculations showed that physical DEF for bismuth, gold, and platinum NPs decreased with the increase in voltage from 50 kVp to 100–300 kVp [59]. On the contrary, we found that voltage increase from 50 kVp to 200 kVp enhanced the radiosensitization.

**Figure 10:**
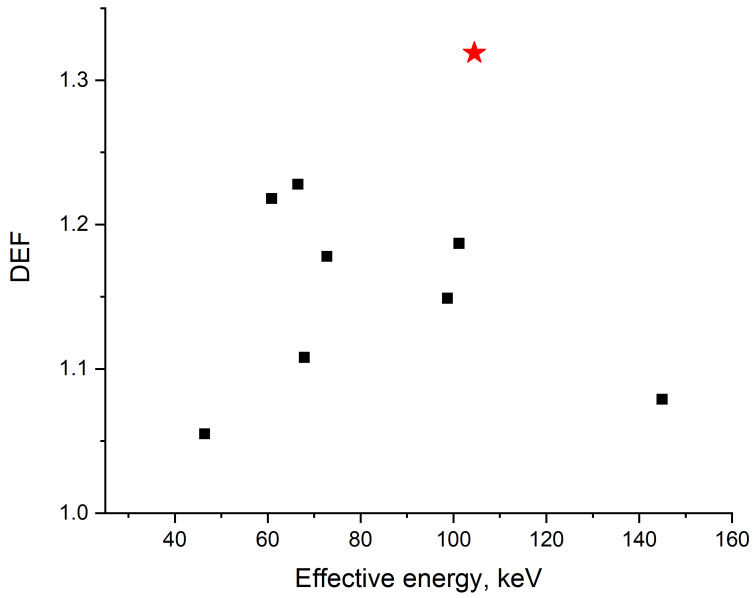
Comparison of calculated physical dose enhancement factor (DEF) for X-ray spectra with various effective energies at a voltage of 200 kVp (black dots, see supplementary files) with DEF for the spectrum of RUST-M1, used in our study (red star).

The magnitude of radiation-induced damage is largely determined by the amount of absorbed photon energy, which depends on the spectral characteristics of the radiation source. We calculated the macroscopic physical increase in the absorbed dose (DEF) for spectra of various X-ray tubes (Table S1) at 200 kVp (Figure 10). For spectra with different effective energies, a significant spread of DEF values was found. The highest calculated DEF (~1.32) value corresponded to the X-ray machine used in the present work. For other cases, DEF values varied from ~1.06 to ~1.23. We found no function to adequately describe the results for this set of X-ray spectra.

Thus, direct comparison of radiosensitization efficacies of NPs obtained with X-ray devices with different spectral properties, even at equal voltage on the X-ray tube, is incorrect. The results obtained with individual X-ray units may vary significantly. Given the variety of experimental devices with different spectral composition, data obtained in each case can be essentially unique. At the same time, the types of X-ray machines used in the clinic are limited, which allows us to hope for data unification.

## 4. Conclusions

In the present study, the search for optimal conditions of orthovoltage X-ray exposure to achieve the greatest effect of radiosensitization by GNPs of various sizes was performed. Experiments with radiation-induced damage of the plasmid DNA identified the X-ray tube voltage, dose rate, and particle size as critical parameters of radiosensitization by GNPs. The greatest efficacy was observed for the largest tested GNP size (26 nm) and the highest voltage (200 kVp). This voltage was found to be optimal for all GNP sizes. However, under a certain dose rate, a more pronounced radiosensitization was detectable for smaller GNPs. Thus, GNPs are an effective tool for increasing the efficacy of the X-ray RT. However, their application requires careful selection of irradiation parameters.

In the present work, the greatest radiosensitization was demonstrated for 2.4 mg/mL GNPs. Although the toxicity of GNPs may vary depending on their design and used biological model, the concentration range of gold seems suitable for both cell models and for an in vivo application [60,61,62,63].

The results obtained are difficult to extrapolate to more complex biological systems since the relationship between physical and biological effects is extraordinarily complex. Due to the biological response to radiation, the situation may vary significantly. Moreover, although some studies have shown an increase in radiation-induced cellular DNA damage in the presence of NPs [64,65], it was shown that cell death may be due to other mechanisms [66,67].

We also note that in in vitro experiments (and, especially, in vivo), the adsorption of protein molecules (as well as other cell compounds, e.g., glucose, amino acids, etc.) on the GNPs’ surface may change the radiosensitization and observed dose rate effect to some extent. Therefore, our further work will be devoted to the complex study of GNPs’ radiosensitization on the panel of cellular models using kV and MV radiation sources with different irradiation conditions.

## Figures and Tables

**Table 1:** Characteristics of gold nanoparticle (GNP) dispersions (*C*_Au0_ = 3 mg/mL).

Sample	GNP Diameter, nm	[GNP], nM	[GNP], mL^−1^
1	12.0 ± 0.5	280	1.7 × 10^14^
2	14.9 ± 0.8	146	8.8 × 10^13^
3	21.0 ± 1.2	52	3.1 × 10^13^
4	26.2 ± 2.0	27	1.6 × 10^13^

**Table 2:** EF values for different voltages on the X-ray tube in the presence of 2.4 mg/mL of 26 nm GNPs.

Dose, Gy	Voltage, kVp		
	100	150	200
5	1.13 ± 0.02	1.17 ± 0.08	1.26 ± 0.09
10	1.27 ± 0.04	1.36 ± 0.09	1.58 ± 0.10
15	1.43 ± 0.05	1.58 ± 0.11	1.98 ± 0.12
20	1.61 ± 0.07	1.84 ± 0.13	2.49 ± 0.13

**Table 3:** Sensitizer enhancement ratio (SER) values for different voltages on the X-ray tube in the presence of 2.4 mg/mL of 26 nm GNPs.

Voltage, kVp		
100	150	200
1.91 ± 0.32	2.17 ± 0.56	2.74 ± 0.61

## Supplementary Materials

The following are available online at https://www.mdpi.com/2079-4991/10/5/952/s1. Figure S1: Normalized spectra calculated using Geant4 for a given set of X-ray tubes. Table S1: Characteristics of X-ray tubes used in the Monte-Carlo simulation.
