# Supplementary material for: Radiosensitization by Gold Nanoparticles: Impact of the Size, Dose Rate, and Photon Energy
Source: Nanomaterials (Basel). 2020 May 17;10(5):952. doi: 10.3390/nano10050952 (PMC7279506; doi:10.3390/nano10050952)
Supplement: Supplementary file 1 [file nanomaterials-10-00952-s001.pdf]

Supporting Information for the manuscript

Radiosensitization by Gold Nanoparticles: Impact of the Size, Dose Rate and Photon Energy

Kirill V. Morozov [1,2], Maria A. Kolyvanova [1,3], Maria E. Kartseva [4], Elena M. Shishmakova [4], Olga V. Dement'eva [4], Alexandra K. Isagulieva [1,5], Magomet H. Salpagarov [1], Alexandr V. Belousov [1], Victor M. Rudoy [4], Alexander A. Shtil [1,6], Alexander S. Samoylov [1] and Vladimir N. Morozov [1,3,*]

[1] State Research Center-Burnasyan Federal Medical Biophysical Center of Federal Medical Biological Agency, 123098 Moscow, Russia; morozov.kv15@physics.msu.ru (K.V.M.); kolyvanova@physics.msu.ru (M.A.K.); kia2303@yandex.ru (A.K.I.); magometonco@mail.ru (M.H.S.); belousovav@physics.msu.ru (A.V.B.); shtilaa@yahoo.com (A.A.S.); asamoilov@fmbcfmba.ru (A.S.S.)
[2] Department of Physics, Lomonosov Moscow State University, 119234 Moscow, Russia
[3] Emanuel Institute of Biochemical Physics, Russian Academy of Sciences, 119334 Moscow, Russia
[4] Frumkin Institute of Physical Chemistry and Electrochemistry, Russian Academy of Sciences, 119071 Moscow, Russia; maryakar@mail.ru (M.E.K.); alena_shishmakova@mail.ru (E.M.S.); redmoun@mail.ru (O.V.D.); dema_ol@mail.ru (V.M.R.)
[5] Gause Institute of New Antibiotics, 119021 Moscow, Russia
[6] Blokhin National Medical Research Center of Oncology, 115478 Moscow, Russia
* Correspondence: morozov.v.n@mail.ru; Tel.: +7-985-117-36-03

Received: 4 April 2020; Accepted: 9 May 2020; Published: date

Table S1. Characteristics of X-ray tubes used in the Monte-Carlo simulation.

Number	Effective energy, keV	Filtration, mm	Anode material
#1	46.39	0.8 Be	W
#2	60.83	0.5 Cu + 2 Al	W
#3	66.48	2 Sn + 4 Al	W
#4	67.89	1.15 Cu + 4 Al	W
#5	72.72	0.99 Cu + 6 Al	W
#6	98.71	1 Cu + 4 Al	W
#7	101.19	1.2 Cu + 4 Al	W
#8	104.50	1.5 Al	W
#9	144.92	1.0 Pb + 3.0 Sn + 2.0 Cu + 4.0 Al	W

#8 - stands for the set of characteristics of RUST-M1 machine, used in our study.

#1 - 46.39 keV

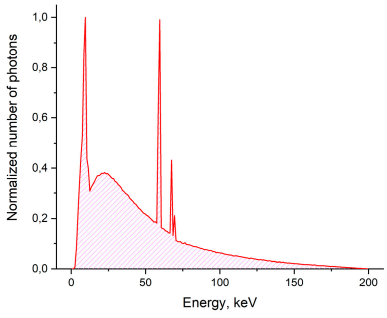

#2 - 60.83 keV

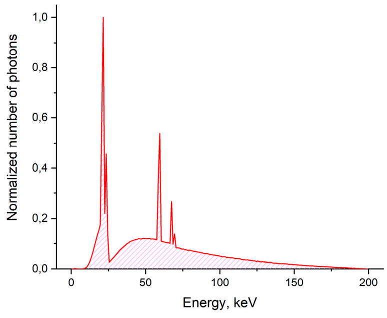

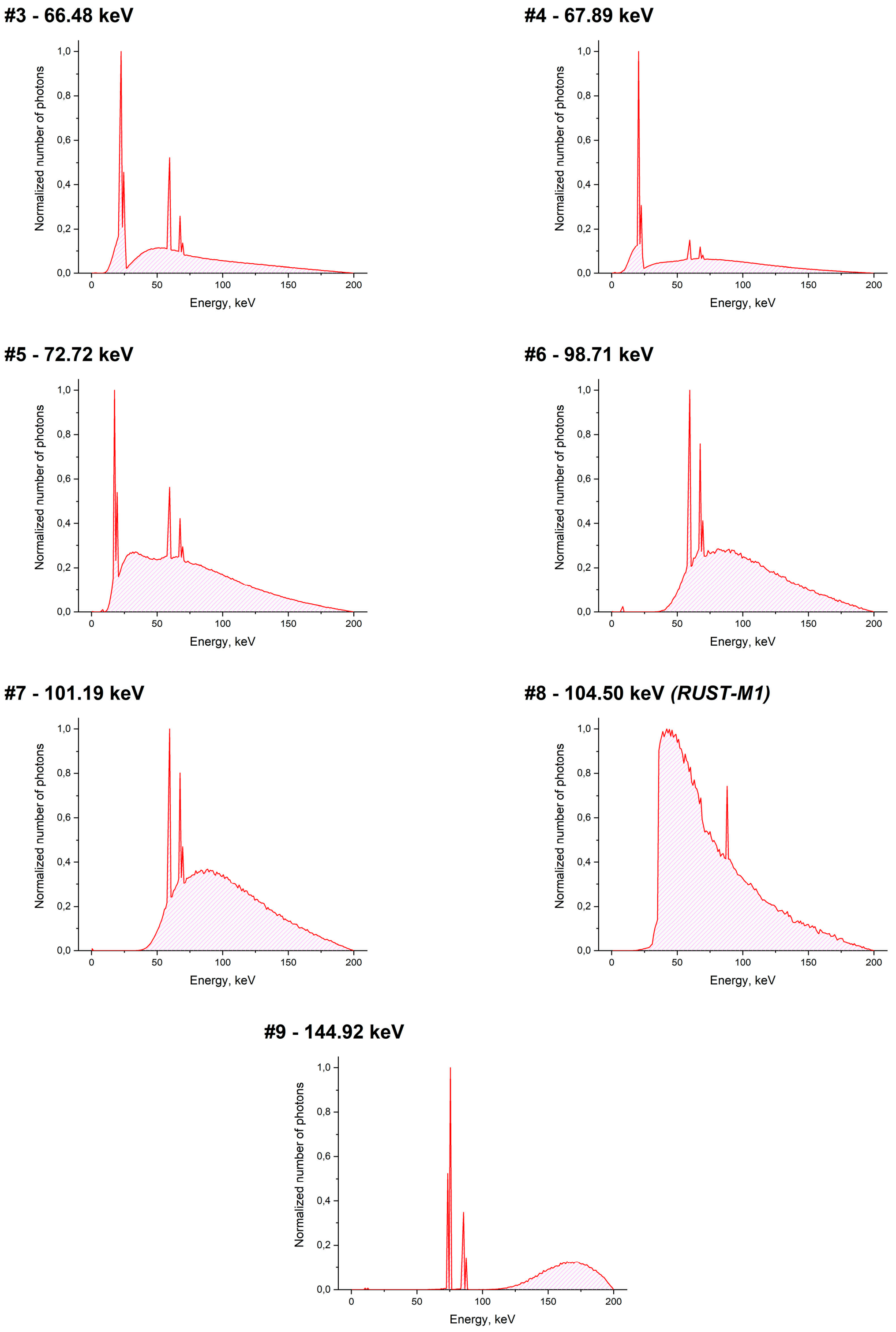

Figure S1. Normalized spectra calculated using Geant4 for a given set of X-ray tubes. #8 is the spectrum of RUST-M1 spectrum, used in our study.
